# Design, Characterization, and In-Vitro Targeted Delivery of Melatonin via pH-Responsive Niosome Nanoparticles Decorated with Folate Against Colon Cancer Cells

**DOI:** 10.34172/apb.46393

**Published:** 2026-06-01

**Authors:** Davoud Jafari-Gharabaghlou, Urjwan Alali, Fadhil Jawad Al-Tu’ma, Hamid Babavalian, Mozafar Mohammadi, Mohammad Sadegh Hashemzadeh

**Affiliations:** ^1^Nanobiotechnology Research Center, New Health Technologies Institute, Baqiyatallah University of Medical Sciences, Tehran, Iran; ^2^Department of Pharmaceutical Chemistry, College of Pharmacy, University of Kerbala, Kerbala, Iraq; ^3^Medical Laboratories Techniques Department, College of Health and Medical Techniques, Al-Mustaqbal University, 51001, Babylon, Iraq; ^4^Department of Chemistry and Biochemistry, College of Medicine, University of Kerbala, Kerbala, Iraq; ^5^Applied Biotechnology Research Center, New Health Technologies Institute, Baqiyatallah University of Medical Sciences, Tehran, Iran

**Keywords:** Melatonin, Colorectal cancer, Targeted therapy, Niosome, Folic acid

## Abstract

**Purpose:**

Utilizing targeted drug delivery in oncology represents a valuable strategy to enhance the therapeutic efficacy of chemotherapy and simultaneously mitigate its adverse effects. Our present investigation focused on harnessing the therapeutic properties of melatonin as an anti-cancer agent and improving its bioavailability by employing a drug delivery system based on folic acid-decorated niosomal nanoparticles.

**Methods:**

To deliver melatonin, a niosomal and a folic acid decorated niosomal system was used. Nanoparticles were synthesized with the thin film hydration method and characterized by AFM, SEM, DLS, and FT-IR techniques. The cytotoxic effects and changes in gene expression were assessed using MTT and real-time PCR techniques, respectively. Additionally, flow cytometry experiments were conducted to investigate their impact on cell cycle arrest and apoptosis induction.

**Results:**

Nanoparticle analysis revealed that the size distribution of blank niosomes, melatonin-loaded niosomal nanoparticles, and melatonin-loaded folic acid-decorated niosomal nanoparticles were 157±8.96 nm, 198±12.34 nm, and 239.9±39.64 nm, respectively. Controlled release studies indicated that over 96 hours, the maximum amount of melatonin released was approximately 60% at pH 7.4 and 73% at pH 5. The results demonstrated enhanced cell cytotoxicity of melatonin when encapsulated in melatonin-loaded folic acid-decorated niosomal compared to pure melatonin and niosomal nanoparticles. Furthermore, melatonin-loaded folic acid-decorated niosomal nanoparticles exhibited superior outcomes in terms of promoting apoptosis and cell cycle arrest compared to other tested substances.

**Conclusion:**

Overall, melatonin-loaded folic acid-decorated niosomal nanoparticles demonstrate a significant capability as an effective therapeutic strategy for targeting colorectal cancer cells.

## Introduction

 The third most frequently diagnosed malignancy is colorectal cancer (CRC), which also stands as the second leading factor in cancer-related fatalities on a global scale. Roughly 2.2 million new CRC cases were identified in 2023, resulting in more than 900,000 deaths.^1,2^ Based on projections of human development, population growth, and aging, the incidence of new cases of CRC worldwide is expected to reach 3.2 million by 2040.^3^ The primary approach utilized in CRC therapy to extend the life cycle of patients is chemotherapy.^4^ The present chemotherapy treatments involve using either a single drug, predominantly fluoropyrimidine (5-FU), or combining one or more drugs such as oxaliplatin (OX), capecitabine (CAP), and irinotecan (IRI) in various regimens.^1^ Nevertheless, the advantages of chemotherapy are frequently restricted by its toxicity, acquired resistance, side-effects, low tumor-specific selectivity, and unsatisfying response rate.^4^ Different genes and protein expressions are involved in the cell cycle and apoptosis of cells. The regulation of apoptotic cell death is heavily influenced by the Bcl-2 protein family, and in many cases, the dysregulation of these proteins can be linked to cancer.^5^ Among the members of this family, Bcl-2 has been extensively researched within the realm of cancer studies. While its initial role was identified as a primary regulator of apoptosis, subsequent research revealed that Bcl-2 is also involved in activities such as cell migration, invasion, and the spread of tumors.^6^ The protein Bax, which also belongs to the Bcl-2 family, has a critical function in inducing apoptosis in cancer cells. This protein aids in controlling the apoptosis mechanism in both healthy and malignant cells.^7^ The human telomerase reverse transcriptase, also known as hTERT, is a vital component in the process of cancer apoptosis. It serves as the principal subunit of the telomerase core and plays an indispensable role in preserving chromosome stability and integrity. Studies have shown that preventing the activity of hTERT can significantly hinder proliferation and prompt apoptosis, indicating its potential use as a target in cancer treatment.^8^ The protein known as Cyclin D1 has a significant impact on the regulation of invasion, adhesion, cell cycle, and tumor-stroma interactions in different cancer varieties. It is recognized as a driving force behind the development of cancers, and when it becomes dysregulated, it leads to the progression of tumors and metastasis. Researchers have noted an increase in Cyclin D1 level across several tumor types, and this increase is viewed as an unfavorable predictor of the disease’s outcome.^9^ The inefficiencies of chemotherapy have led researchers to use non-harmful and natural substances instead of common chemotherapy drugs in cancer treatment. Melatonin (Mel) is one of these substances that has recently attracted the researcher’s attention. Mel is predominantly created by the pineal gland, and regulates the circadian rhythm and facilitates physiological adaptations in response to the day-night cycle.^10^ Extensive research has demonstrated that melatonin can effectively decrease the development and advancement of cancer by acting as a powerful antioxidant. This is achieved through its ability to scavenge radicals directly or indirectly by stimulating the antioxidant enzyme expression.^11,12^ However, the clinical applications of Mel face limitations because of its low solubility in aqueous media. Melatonin is sparingly soluble in water, with a solubility of 0.1 mg/mL at room temperature.^13,14^ This leads to a very short half-life (45 min) and limited bioavailability in blood circulation.^10^ Nowadays, the advancement of nanotechnology offers fresh possibilities for treating CRC.^4^ Encapsulating anticancer drugs can offer several benefits, such as modifying their bio-distribution, enhancing solubility and bioavailability, and enabling easier entry into cancer cells.^15^ Numerous types of nanoparticles (NPs), such as dendrimers, liposomes, polymeric micelles, mesoporous silica particles, and niosomes, have been used to carry different categories of therapeutic agents, which include antiangiogenic agents, cytotoxic agents, small-interference RNA, and chemosensitizers.^16,17^ Niosomes have become a subject of growing scientific interest in the nanotechnology field since they offer distinctive versatility as effective drug delivery systems for various therapeutic applications.^18^ Niosomes, which are also known as nonionic surfactant vesicles, are created using a combination of cholesterol and nonionic surfactants. They can form from various hydrophilic head groups and one to two hydrophobic alkyl groups, which allows them to entrap both hydrophilic and hydrophobic molecules within their structure.^19^ Niosomes enhance how the drug exerts its pharmacological action by decreasing the amount of medication utilized, enhancing the solubility of poorly soluble medicines, protecting the drug from the biological environment, delaying the clearance from the circulation, and restricting their effects only to the target cells.^20^ Folic acid has attracted much attention as a targeting ligand due to its high affinity for folate receptors, which are overexpressed on the surface of many types of cancer cells, including colorectal cancer (SW480 (high expression)).^21^ This specific binding facilitates better delivery of folic acid-functionalized nanocarriers to malignant cells while minimizing their entry into normal tissues. Although folic acid has been widely employed in polymer- and lipid-based drug delivery platforms due to its selective recognition of folate receptors on cancer cells, its attachment to covalent organic frameworks for targeted therapeutic applications remains relatively uncommon and represents a nascent area of ​​research.^22,23^

 Targeted therapies can impede cancerous cells from proliferating, differentiating, and migrating by directly targeting them.^1^ Niosomal (Nio) NPs could be decorated with different substances like folic acid or hyaluronic acid and used in targeted drug delivery for cancer cells.^24-26^ In the current study, we utilized melatonin as an anti-cancer agent and enhanced its bioavailability using a folic acid decorated niosomal (Fol-Nio) NPs drug delivery system for targeted delivery against colon cancer cells.

## Material and Methods

###  Preparation of Folic Acid-Conjugated Niosomal NPs

 The thin-film hydration technique was used to prepare niosome nanoparticles (Nio-NPs) and folic acid-conjugated niosome nanoparticles (Fol-Nio). Briefly, a solution of 6 mg cholesterol and 36 mg Span 60 was dissolved in 10 mL of a 1:1 (v/v) chloroform/methanol mixture and transferred to a 25 mL round-bottom flask. The solvent was then evaporated under vacuum at 55°C with stirring at 150 rpm for 60 min, resulting in the formation of a thin film on the inner surface of the flask. Fol-Nio NPs were then synthesized by incorporating 4.4 mg folic acid and hydrating the thin film with 10 mL phosphate-buffered saline (PBS) at 50°C. Ultrasonication was used to homogenize the samples and achieve an optimal particle size distribution. To prepare folic acid-decorated niosomal nanoparticles containing melatonin (Fol-Nio/Mel), the same protocol was followed, just in the initial step, 23 mg of melatonin was added to the chloroform/methanol solution. Similarly, Nio NPs and Nio/Mel NPs were prepared using the same methods and amounts, except that folic acid was omitted from the synthesis.

###  Characterization of Niosomal Nps

 A benchtop dynamic light scattering system (DelsaMax Pro, Beckman Coulter) was utilized at 25°C to measure the zeta potential, particle size, and polydispersity index (PDI) of the nanoparticle formulations. The samples were freeze-dried using a FreeZone Freeze Dry System (Labconco). Additionally, the morphology and shape of the samples were analyzed by atomic force microscopy (AFM) with a MultiMode AFM (Bruker), transmission electron microscopy (TEM) with a Talos TEM (Thermo Fisher Scientific), and scanning electron microscopy (SEM) with a GeminiSEM (Carl Zeiss). To investigate the molecular interaction between melatonin, Span 60, cholesterol, folic acid, and the fabricated NPs, Fourier Transform Infrared Spectroscopy (FT-IR) (Spectrum Two, USA) was utilized. The FT-IR analyses were conducted at room temperature over a scanning range of 4000 to 400 cm⁻¹ with a constant resolution of 4 cm⁻¹.

###  In Vitro Melatonin Release

 The in vitro release profile of melatonin was studied using the dialysis bag diffusion method. In short, 5 mL of the Fol-Nio/Mel formulation was loaded into a semi-permeable dialysis membrane (cutoff molecular weight: 12 kDa, cellulose acetate) and immersed in 60 mL PBS. The in vitro release study was performed using two pH: 7.4 and 5.0, at a constant temperature of 37 °C while maintaining magnetic stirring (70 rpm) for 72 hours. At pre-defined time points, 1 mL of the release medium was removed and replaced with an equal volume of fresh PBS to maintain sink conditions.

 The release quantity of melatonin was measured to have the absorbance at 277 nm by using UV–Visible spectrophotometer (Cary 60, Agilent Technologies). The release profile of free (non-encapsulated) melatonin in a similar experimental design and at the same drug concentrations served as comparison. Moreover, melatonin release from Nio formulation was also evaluated in parallel with the same protocol.

###  Cell Culture

 The SW480 cells were cultured in RPMI-1640 medium (Sigma, Germany) containing 10% fetal bovine serum (FBS; Biochrom, UK), and 1% penicillin-streptomycin (Sigma, Germany). Cells were cultured at 37 °C in a humidified atmosphere with 5% CO₂. Once the cultures reached ~85–95% confluency, culture medium was removed and the cells were processed for further experiments.

###  Cytotoxicity Test

 The cytotoxicity of the formulations was measured by MTT assay. SW480 cells were plated to a density of 5 × 10³ cells per well in 96-well plates and incubated for 24 hours at 37° C in humidified atmosphere containing 5% CO₂. Free melatonin, Nio/Mel, or Fol-Nio/Mel at concentrations of 0.5, 1, 1.5, 2, 2.5, and 3M and Fol−Nio/Mel (0 to 4mm) were then treated to the cells for 48 hours. After the treatment, MTT solution (2 mg/mL; Alfa Aesar, USA) was added into wells incubated in a dark for further 4 hours. The culture medium was gently removed and 100 μL of dimethyl sulfoxide (DMSO; Fisher Scientific, USA) were dissolved formazan crystals. The plates were shaken gently for 10 min and absorbance was measured at 570 nm by microplate reader. Cell viability was expressed in % as the ratio to untreated control cells. Note: All experiments were performed as the standard MTT assay protocol.

###  Analysis of mRNA Levels 

 Total RNA was isolated from approximately 1 × 10⁶ cultured cells using the RNX-PLUS reagent (Sinagen, Tehran, Iran), adhering to the manufacturer’s protocol. A spectrophotometer then confirmed the quality and concentration of the RNA. For cDNA synthesis, 1 μg of RNA was reverse transcribed using the AccuPower PCR PreMix kit (Bioneer, Daedeok-gu, Daejeon, Korea). Quantitative real-time PCR (qRT-PCR) was then carried out on a micPCR system (Bio Molecular Systems, Australia) using SYBR Premix Ex Taq (Takara Bio, Otsu, Shiga, Japan) according to the supplier’s recommendations. Relative gene expression levels were analyzed using the 2^−ΔΔCt method, with a housekeeping gene serving as an internal control. The RT-PCR primers employed were Bax F 5’- AGTGTCTCAAGCGCATCGG-3’, R 5’- TGCAGCTCCATGTTACTGTCC-3’, Bcl-2 F 5’- CCACAAGTGAAGTCAACATGC-3’, R 5’- AGTAAATAGCTGATTCGACGTT-3’, hTERT F 5’- TTTATGTCACGGAGACCACGTT-3’, R 5’- TGCTCCAGACACTCTTCCGGTA-3’, cyclin D1 F 5’- CACTTTCAGTCCAATAGGTGT-3’, R 5’- TTACATAGCCAAGATGTGCAA-3’, and GAPDH F 5’- GGTCATCACTATTGGCAACG-3’ and R 5’- ACGGATGTCAACGTCACACT-3’.

###  Cellular Apoptosis Analysis

 Flow cytometry instrument was used to assess cell apoptosis. Briefly, SW480 cells (2 × 10^5^) were seeded into 6-well plates and incubated for 24 hours to ensure proper attachment. The cells were then treated with pure melatonin and melatonin-loaded nanoparticles at their respective IC₅₀ concentrations for 48 hours. After treatment, the cells were harvested, washed twice with cold PBS, and centrifuged at 150 × g for 5 minutes. The resulting pellets were resuspended in 200 µL of binding buffer, followed by the addition of 10 µL of propidium iodide (PI) and 10 µL of FITC-conjugated Annexin V (final concentration: 0.25 µg/mL). The samples were incubated in the dark at room temperature (24 °C) for 20 minutes before flow cytometric analysis. Finally, 200 µL of binding buffer was added, and a flow cytometry device (CytoFLEX, Beckman Coulter, USA) was employed to evaluate the apoptosis.

###  Cell Cycle Assessment

 Initially, SW480 cells (2 × 10^5^ per well) were seeded into 6-well plates and allowed to adhere for 24 hours. Following attachment, the cells were treated with the IC₅₀ concentrations of free melatonin, Nio/Mel, Fol-Nio/Mel for 48 hours. Following treatment, the cells were thoroughly rinsed three times with PBS and subsequently detached using trypsin. For fixation, 70% ethanol was added to the cell suspension, which was then stored overnight at −20 °C. On the following day, cells were collected by centrifugation and resuspended in PBS containing RNase A (1 μg/mL) and PI (100 μg/mL). The mixture was incubated at 37 °C for half an hour to allow staining. Cell cycle analysis was then performed by measuring fluorescence intensity using a flow cytometer equipment (CytoFLEX, Beckman Coulter, USA).

###  Statistical Analysis

 All statistical analyses and graphical displays were performed using GraphPad Prism version 8 (California, USA). Each experiment was performed in triplicate, and data are presented as mean ± standard deviation (SD). Comparisons between two groups were performed using the Student’s t-test, while differences between more than two groups were assessed by one-way analysis of variance (ANOVA). Statistical significance was determined at a threshold of p < 0.05.

## Results and Discussion

###  Characterization of Niosomal NPs

 Niosomes offer great potential as a drug delivery system in cancer therapy. They aid precisely targeting the medication to cancer cells, consequently prolonging treatment duration while minimizing severe side effects and enhancing drug stability. By decreasing particle size and augmenting the amount of trapped drug within niosomal vesicles, the cytotoxicity of the medication against cancer cells is improved.^27^ The present investigation involved the formulation of niosomes utilizing a thin film hydration approach, which is regarded as the optimal, uncomplicated, and consistent technique for creating multilamellar non-ionic niosomal vesicles. Typically, this method is coupled with sonication to obtain niosomes with a narrow size.^17^ Preparing niosomal NPs with a small particle size was a key priority in this study, given that the size of NPs plays a significant role in its physical stability, cellular absorption, and biological distribution. Given that NPs measuring 70-200 nm maintain stability within the bloodstream and possess prolonged circulation properties, synthesized NPs of similar sizes can serve as effective tools for intelligent drug delivery in the pharmaceuticals field, as supported by prior research findings. Furthermore, studies have shown that NPs with a size smaller than 200 nm can bypass the immune system, whereas those under 70 nm often accumulate in the liver.^28^ Figure 1 illustrates the zeta potential and size analysis of Fol-Nio/Mel NPs. The peak in Figure 1A is centered around 239.9 nm, with a PDI of 0.714. The PDI value indicates a moderately polydisperse sample, as values closer to zero correspond to a more monodisperse distribution. Figure 1B illustrates the zeta potential distribution of the nanoparticles, showing a single peak centered at -27.8 mV, which reflects the surface charge of the nanoparticles.

**Figure 1:**
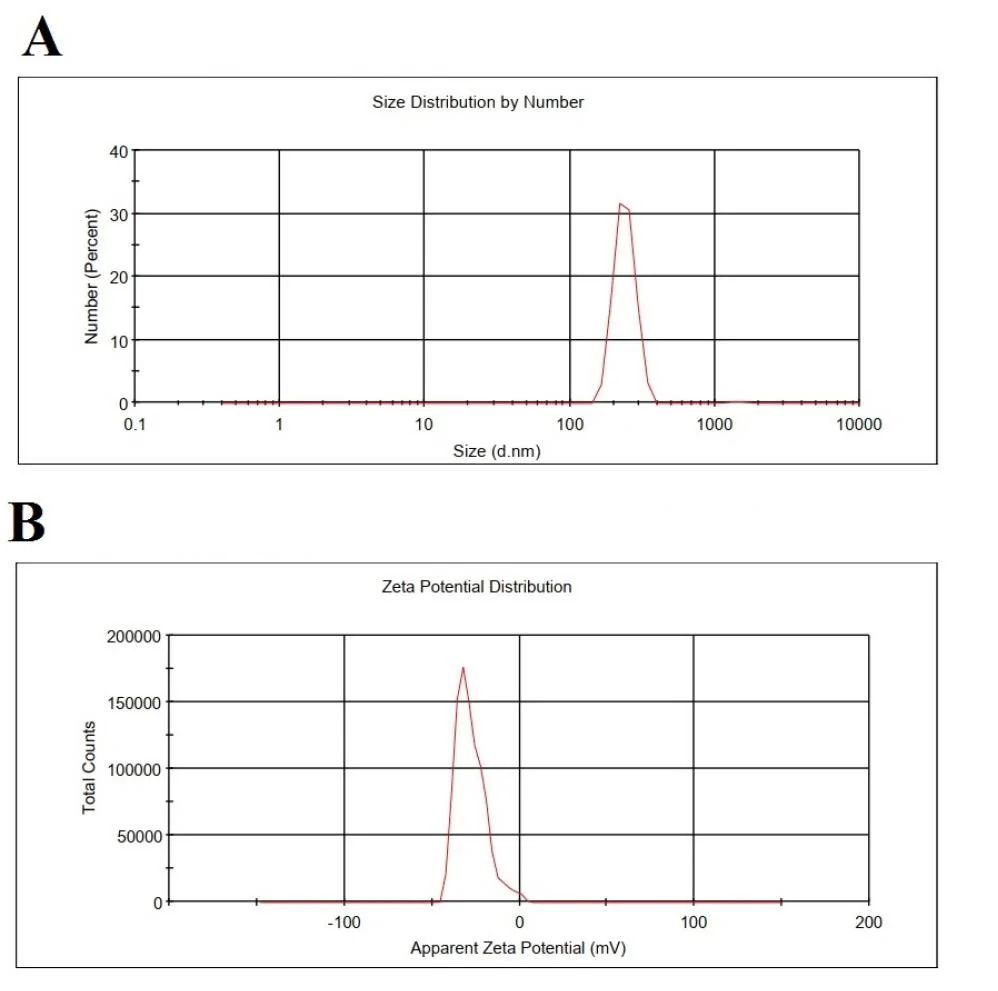
The size (A) and zeta potential (B) result of melatonin-loaded folic acid-decorated niosome nanoparticles by DLS

 The surface charge, size, and PDI of the synthesized niosomal NPs are shown in Table 1. The increased size of Fol-Nio/Mel compared to other NPs, is due to the melatonin load between their two membrane layers and the presence of folic acid on its surface.

**Table 1:** DLS analyses of blank and melatonin loaded niosomal nanoparticles.

**Groups**	**Particle size (nm)**	**Polydispersity Index**	**Zeta potential (mV)**
Niosome	157 ± 8.96	0.648	−19.8 ± 6.
Niosome-Melatonin	198 ± 12.34	0.582	−22.4 ± 5.2
FA- Niosome-Melatonin	239.9 ± 39.64	0.714	−27.8 ± 8.4

 The performance of drug delivery vehicles can be greatly influenced by the shape and morphology of nanoparticles. Specifically, while spherical nanoparticles are known for their stability during circulation, non-spherical nanoparticles offer potential advantages in targeting specific tissues, although they may exhibit reduced stability.^29,30^ According to SEM and TEM results, Fol-Nio/Mel has larger size compared to other NPs which is in that is consistent with the findings of DLS (Figure 2A, Figure 2B, and Figure 2C). Furthermore, according to the findings obtained from AFM analysis (Figure 2D), the emergence of particles above the surface can be ascribed to the existence of Fol-Nio/Mel. It is worth noting that the dimensions recorded through AFM were marginally smaller compared to those derived from the DLS technique. This variation can be attributed to the utilization of dehydrated samples of Fol-Nio/Mel during the imaging procedure.

**Figure 2:**
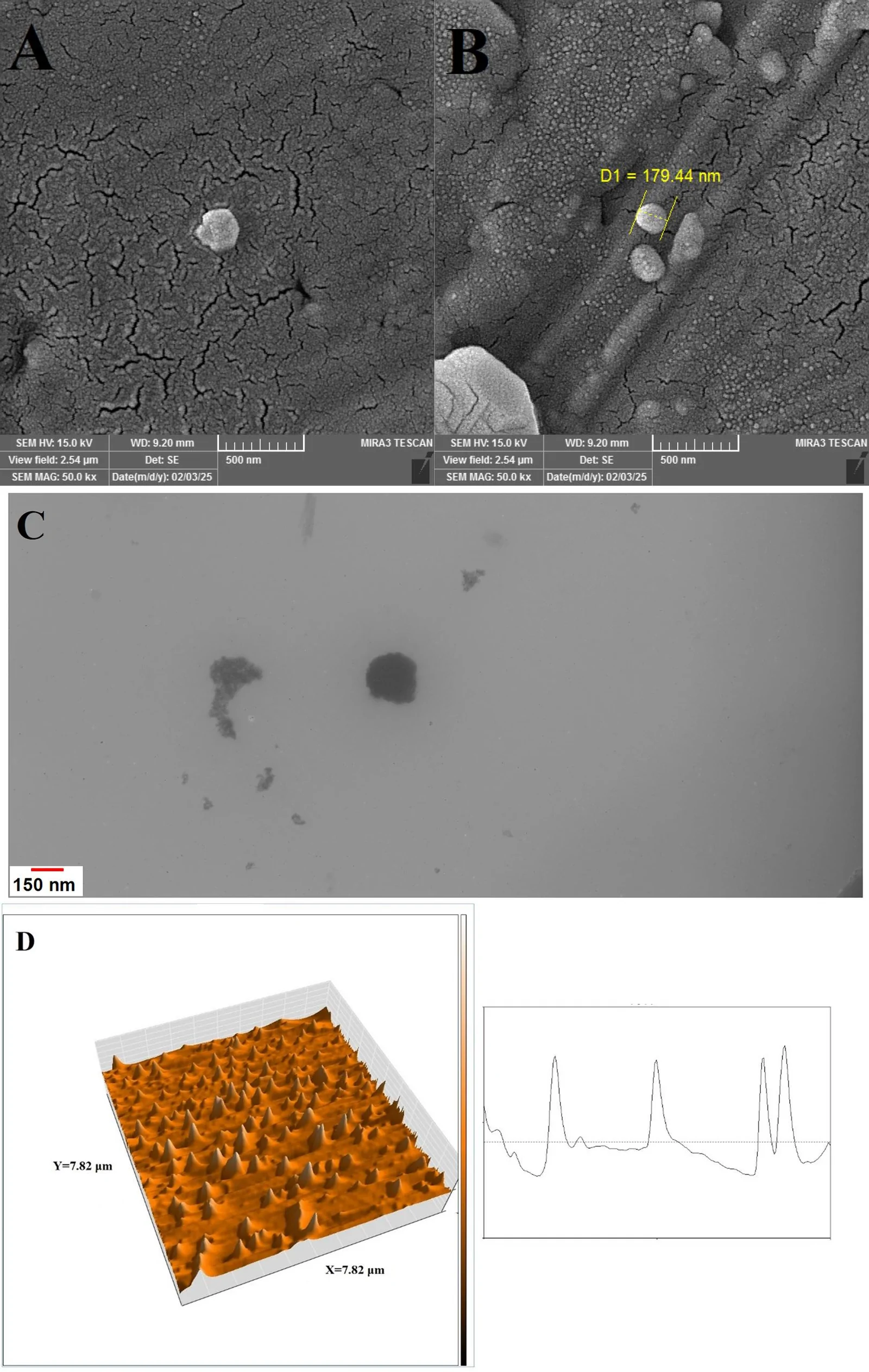
A: SEM Result of melatonin loaded niosome, B: SEM Result of melatonin loaded folic acid-decorate niosome, C: TEM result of melatonin-loaded folic acid-decorated niosome, and D: AFM result of melatonin loaded folic acid-decorate niosome

 The chemical structure of the prepared NPs was investigated using FT-IR analysis. Additionally, the interaction between the synthesized niosomal system and melatonin was assessed. The FT-IR analysis involved monitoring expected peaks associated with Span 60, cholesterol, folic acid, Mel, Nio, Nio/Mel, Fol-Nio, and Fol-Nio/Mel. The presence of these peaks suggested successful loading of melatonin into the niosomal system (Figure 3). The FT-IR spectrum of Span 60 revealed characteristic absorption bands, notably at 1728 cm⁻¹, corresponding to C = O stretching, and at 1167 cm⁻¹, likely attributed to ester functional groups. Symmetric and asymmetric stretching of aliphatic C–H bonds were evident at 2906 cm⁻¹ and 2850 cm⁻¹, respectively, while a peak at 711 cm⁻¹ indicated CH₂ rocking vibrations.^31^ Additionally, broad absorption near 3385 cm⁻¹ was associated with O–H stretching, and another band at 2946 cm⁻¹ confirmed –CH stretching. A pronounced peak at 1726 cm⁻¹ indicated the presence of an ester carbonyl group.^32^ Folic acid exhibited a distinct C = O stretching band around 1649 cm⁻¹. The FT-IR profile of cholesterol displayed typical signals including methylene rocking at 802 cm⁻¹, C–O stretching at 1055 cm⁻¹, C–H stretching within the 2810–3010 cm⁻¹ region, and C–H bending at 1376 cm⁻¹. A broad band observed between 3110 and 3610 cm⁻¹ was attributed to –OH stretching. The FT-IR analysis of the synthesized niosomal nanoparticles showed a notable absorption peak between 3000–3700 cm^−1^, suggestive of strong hydrogen bonding interactions most likely between the hydroxyl groups of cholesterol and Span 60 corroborating earlier reports.^33^

**Figure 3:**
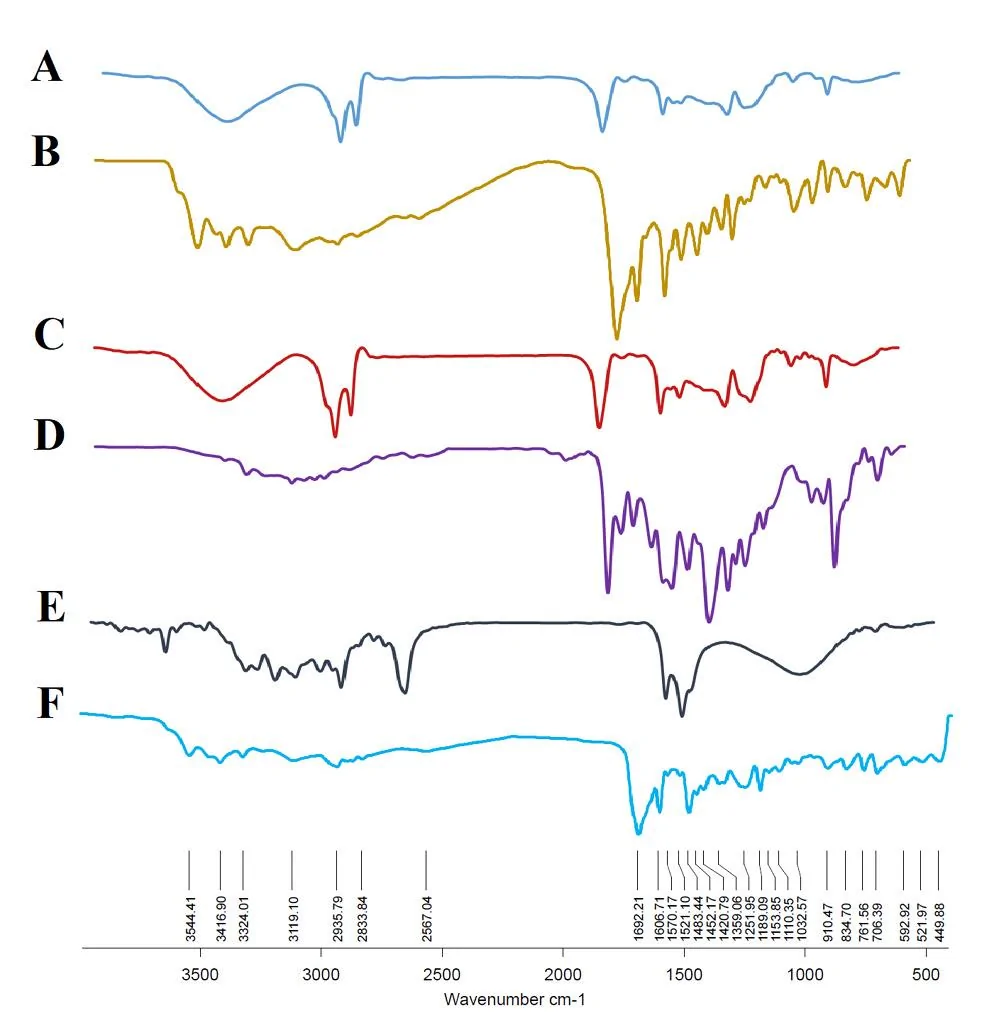
FT-IR spectrum of Span 60 (A), folic acid (B), niosome (C), melatonin (D), melatonin-loaded niosome (E), and melatonin-loaded folate-decorated niosome (F). The results showed that the melatonin was efficiently encapsulated in niosomal nanoparticles

Figure 4, presented the findings of melatonin release from the prepared nanoparticles (Nio/Mel and Fol-Nio/Mel). The release of melatonin was examined over a period of 96 hours in a PBS medium, simulating both physiological conditions with a pH of approximately 7.4 and cancerous pathological conditions with a pH of 5. The results illustrate that the release of melatonin exhibited a biphasic pattern. Initially, there was a rapid burst release phase lasting 12 hours, followed by a subsequent phase characterized by a constant or slower rate of release. This slower release phase occurred after the drug diffused from the outer layers of the niosomes. Importantly, it was observed that the release rates of melatonin from NPs under physiological pH conditions (~7.4) were significantly lower compared to those under cancerous pathological pH conditions (~5). This indicates that the drug release process was influenced by the pH level, demonstrating its pH-dependence.

**Figure 4:**
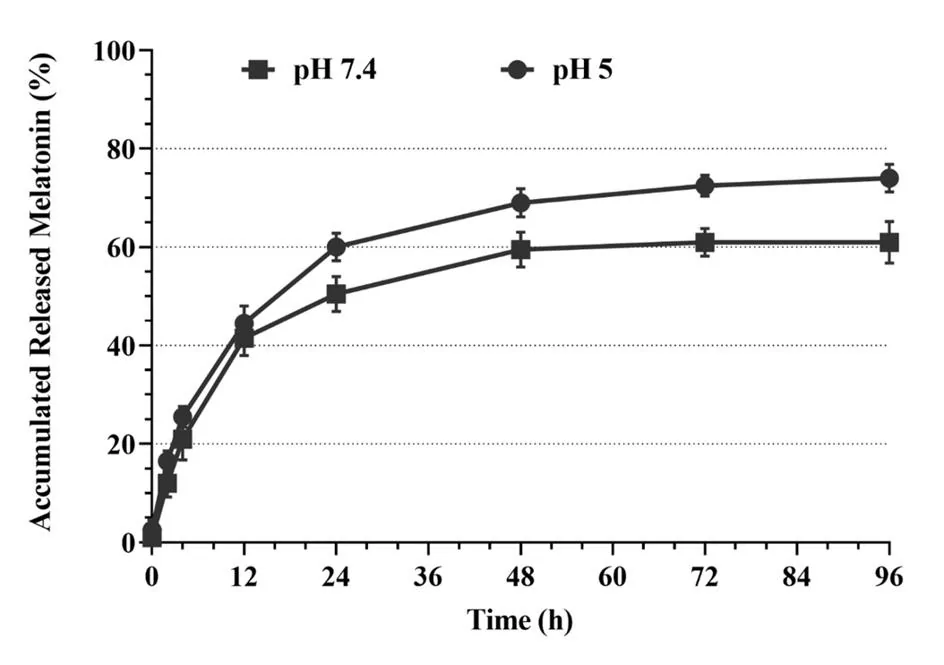
In vitro melatonin release from niosome and folate-decorated niosome nanoparticles at pH 7.4 and pH 5 (37 °C)

 The MTT method was used to evaluate cell cytotoxicity in this study. SW480 cells were treated with pure melatonin, Nio, Nio/Mel, Fol-Nio, and Fol-Nio/Mel for 48 hours at various concentrations. The findings demonstrated that Fol-Nio/Mel and Nio/Mel induced more cell death than pure melatonin in SW480 colon cancer cells during the experiment, whereas Nio and Fol-Nio did not exhibit any notable cytotoxic effects (Figure 5). Consequently, the utilization of nanocarriers enhanced the cytotoxicity of melatonin against SW480 cells in a dose-dependent manner. The IC50 values for pure melatonin, Nio/Mel, and Fol-Nio/Mel were found to be 1.91 mM, 1.39 mM, and 1.02 mM respectively, leading to the selection of this concentration for subsequent tests. Moreover, the survival rate of over 90% of cells treated with Nio and Fol-Nio indicated minimal lethal effects on the cells. As a result, with no significant difference found between the control group, the Nio treated group, and Fol-Nio treated group, prompting the exclusion of this treatment group from further experiments. At low concentrations (0-1 mM), melatonin shows a slight decrease in cell viability, but the effect is not statistically significant (ns). However, at higher concentrations (1.5-4 mM), melatonin exhibits a more pronounced decrease in cell viability, with statistically significant differences compared to the control (0 mM). Niosome encapsulation significantly enhances the cytotoxic effect of melatonin. Even at low concentrations (0.5-1 mM), a noticeable reduction in cell viability is observed. The effect becomes more prominent at higher concentrations, showing a dose-dependent decrease in cell viability. Folate conjugation further enhances the cytotoxic effect of niosome-encapsulated melatonin. This suggests that folate targeting may improve the cellular uptake of the nanoparticles, leading to increased cytotoxicity. The results demonstrate that niosome encapsulation significantly enhances the cytotoxic effect of melatonin. Folate conjugation further improves the efficacy of the nanoparticles, likely due to enhanced cellular uptake. This study suggests that folate-conjugated niosome-encapsulated melatonin could be a promising therapeutic strategy for cancer treatment.

**Figure 5:**
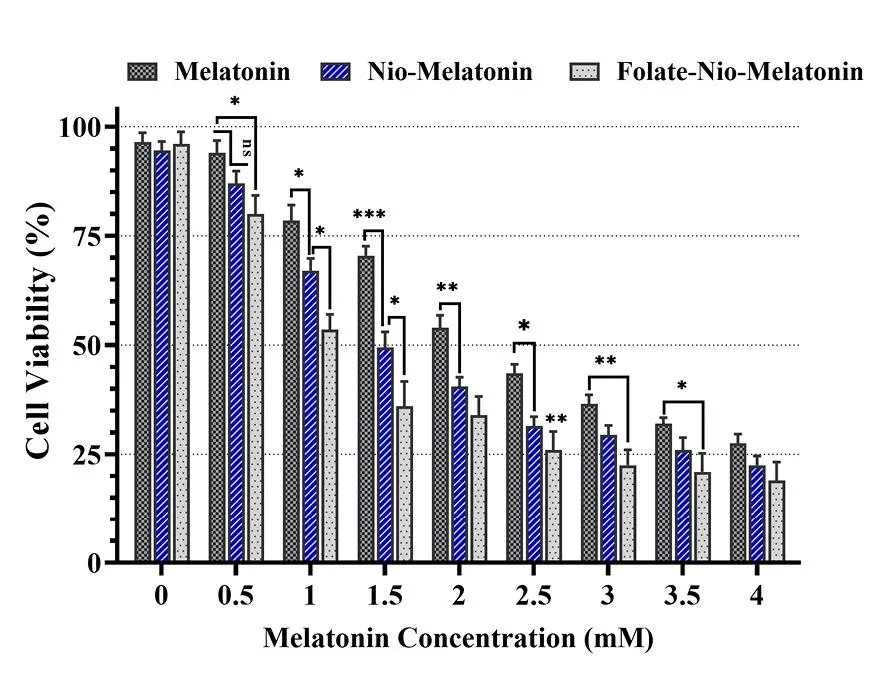
MTT assay demonstrated the in vitro cytotoxic effects of pure melatonin, melatonin-loaded niosome, and melatonin-loaded folic acid-decorated niosome on SW480 colon cancer cells

 To investigate the effect of pure melatonin, Nio/Mel, and Fol-Nio/Mel on SW480 cancer cells, Bax, Bcl-2, hTERT and cyclin D1 genes expression were studied. Bax functions as a protein that promotes programmed cell death, known as apoptosis.^34^ When Bax is activated, it triggers cell death, whereas when it is deactivated, it can make tumor cells less responsive to apoptosis and contribute to the development of tumors.^35^ Bcl-2 is a protein that plays a crucial role in regulating programmed cell death in cancer cells. The inhibition of Bcl-2 has been explored as a potential therapeutic strategy for cancer treatment. Targeting the Bcl-2 family of proteins, including Bcl-2, with inhibitors has shown promise in preclinical studies and clinical trials.^36^ The presence of telomerase reverse transcriptase (hTERT) is of utmost importance in the advancement and spread of cancer. The induction of hTERT expression triggers telomerase activation, a prevalent characteristic found in nearly all types of human cancers. This activation, linked to unrestricted cell proliferation, has been extensively studied in clinical research, revealing a direct correlation between elevated hTERT expression, cancer progression, and unfavorable outcomes. By stabilizing telomeres, the activation of telomerase through hTERT significantly contributes to the malignant transformation process.^37,38^ Cyclin D1, a vital protein in controlling the cell cycle, serves a major role in the development of different cancer types. In the case of colorectal cancer (CRC), increased cyclin D1 expression has been linked to improved overall survival and a lower risk of recurrence.^9^ The data suggests that melatonin, particularly when encapsulated in niosomes and conjugated with folate, effectively modulates the expression of cell proliferation-related genes (hTERT, Cyclin D1), survival (Bcl-2), and apoptosis (Bax). FA-Nio-Melatonin consistently exhibits the most significant effects, indicating that folate targeting enhances the efficacy of niosome-encapsulated melatonin. The observed decrease in hTERT, Cyclin D1, and Bcl-2 expression, along with the increase in Bax expression, suggests that these treatments induce apoptosis through inhibition of proliferation and induction of apoptosis. The results of Studies examining the expression of these genes revealed a reduction in the expression of of Bcl-2, hTERT and cyclin D1 genes and an increase in the Bax level (Figure 6), however, these changes were more evident in the therapeutic approach for colorectal cancer cells with Nio/Mel and Fol-Nio/Mel compared to the melatonin, these results indicate the apoptosis induction in cancer cells using the melatonin both in nano-loaded form and pure form.

**Figure 6:**
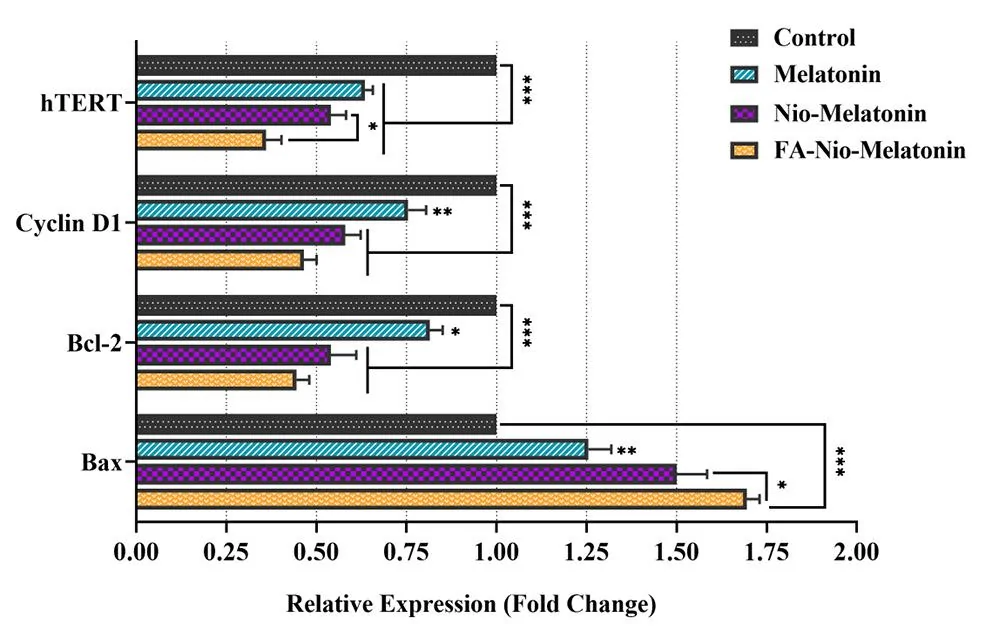
Expression levels of Bax, hTERT, Bcl-2, and cyclin D1 genes in SW480 cells after treatment with pure melatonin, melatonin-loaded niosome, and melatonin-loaded folic acid-decorated niosome

 Following double staining of the cells, flow cytometry was employed to quantitatively measure apoptosis in SW480 cancer cells. Treatment with pure melatonin, Nio/Mel, and Fol-Nio/Mel induced apoptosis in colorectal cancer cells. The percentage of apoptotic SW480 cells significantly increased compared to the control group after exposure to pure melatonin, Nio/Mel, and Fol-Nio/Mel (Figure 7). Notably, Fol-Nio/Mel treatment resulted in a substantially higher percentage of apoptotic cells than either pure melatonin or Nio/Mel alone. These findings are consistent with previous research suggesting that the intracellular release of anticancer drugs from NPs contribute to their cytotoxic effects.^39^ The flow cytometry analysis demonstrates that melatonin, particularly when encapsulated in niosomes and conjugated with folate, effectively induces apoptosis. FA-Nio-Melatonin consistently exhibited the most significant effects, indicating that folate targeting enhances the efficacy of niosome-encapsulated melatonin. The observed decrease in viable cells, along with increases in early and late apoptotic cells, suggests that these treatments primarily induce cell death through apoptosis. The percentage of necrotic cells remained relatively low across all treatments, indicating that apoptosis is the dominant mechanism of cell death. Overall, these results align with gene expression data, which showed that Fol-Nio/Mel more effectively modulated apoptosis-related gene expression than the other groups.

**Figure 7:**
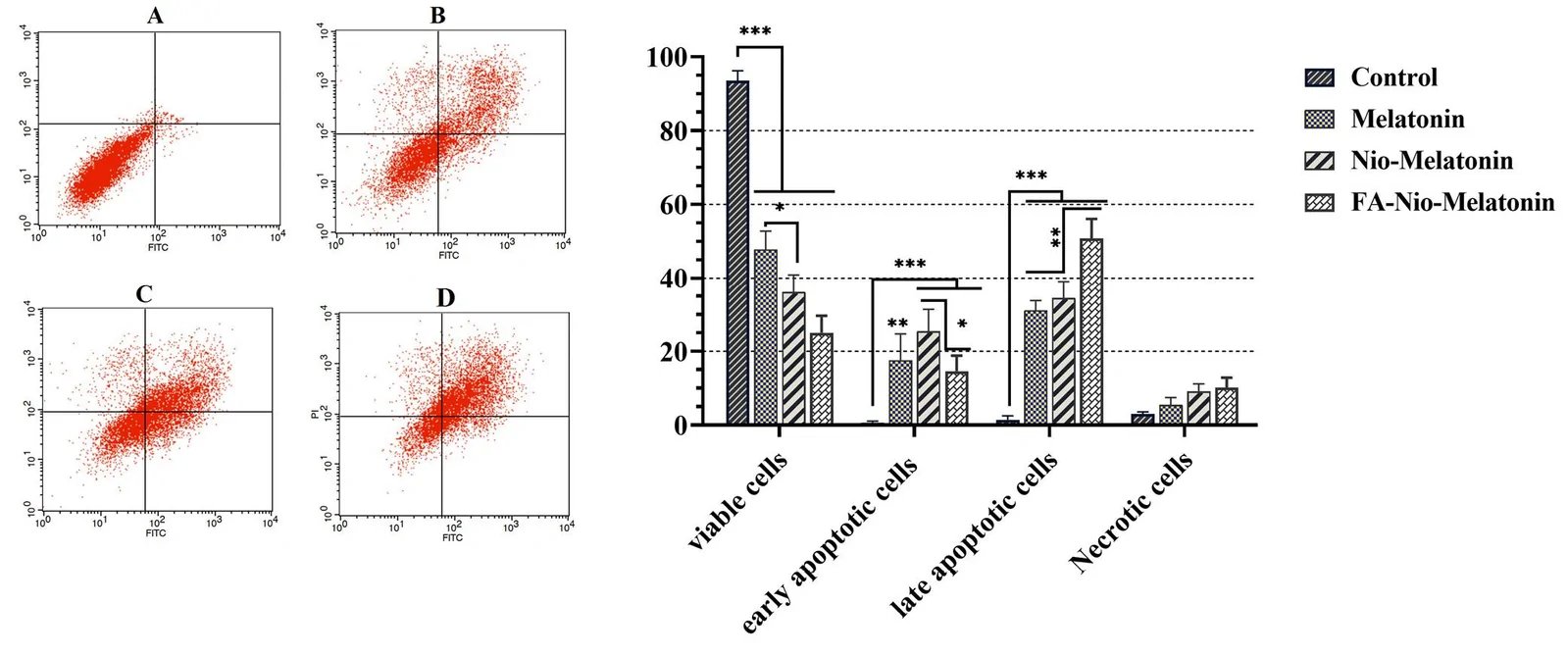
The apoptosis results of the colorectal cancer cells that treated by pure melatonin, melatonin-loaded niosome, and melatonin-loaded folic acid-decorated niosome. (A): Control group, (B): Pure melatonin, (C): Melatonin-loaded niosome nanoparticles, and (D): Melatonin-loaded folic acid-decorated niosome nanoparticles

 Flow cytometry (Figure 8) was used to assess the effects of pure melatonin, Nio/Mel, and Fol-Nio/Mel on cell cycle progression in SW480 cancer cells. The results showed that treatment with these agents induced cell cycle arrest in the sub-G1 phase. Approximately 20% of cells treated with pure melatonin were in the sub-G1 phase, compared to 32% for Nio/Mel and 43% for Fol-Nio/Mel. These findings suggest that melatonin, especially when encapsulated in niosomes and conjugated with folate, effectively disrupts cell cycle progression. Fol-Nio/Mel consistently exhibits the most significant effects, indicating that folate targeting enhances the efficacy of niosome-encapsulated melatonin. The observed increase in the Sub G1 phase and the decrease in the G0/G1, S, and G2/M phases suggest that these treatments induce cell death through apoptosis and inhibit cell proliferation by arresting the cell cycle. This observation aligns with the results of the apoptosis and gene expression studies, which demonstrated a higher impact of Fol-Nio/Mel on cancerous cells.

**Figure 8:**
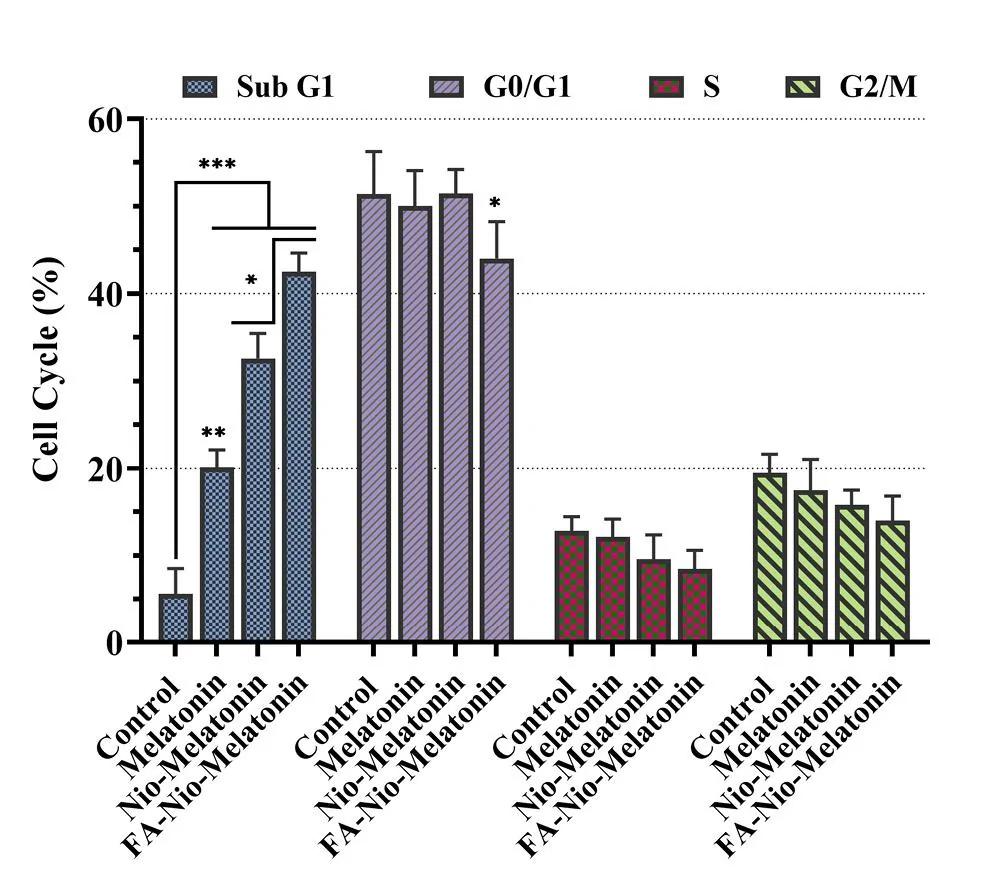
Cell cycle analysis of SW480 cells that treated by pure melatonin, melatonin-loaded niosome, and melatonin-loaded folic acid-decorated niosome

## Conclusion

 The effectiveness of conventional chemotherapy in cancer treatment is often compromised by the emergence of drug resistance. In this study, we investigated the potential of melatonin-loaded niosomes as a novel therapeutic approach against colorectal cancer cells. To enhance targeted delivery, the surface of the niosomal nanoparticles was coated with folic acid to improve selective uptake by cancer cells overexpressing folate receptors. Our findings indicate that niosomal delivery of melatonin resulted in higher expression of apoptosis-related genes and increased apoptosis compared to traditional melatonin treatment, particularly with the folate-decorated niosomal nanoparticles. Additionally, the niosomal carriers we developed demonstrated favorable characteristics in terms of size, zeta potential, PDI, drug loading efficiency, and drug release characteristics. The results suggest that delivering melatonin via niosomal nanoparticles holds potential as an innovative therapeutic strategy for cancer management. However, additional investigations, particularly into clinical models, are essential to fully assess the safety and therapeutic efficacy of this delivery system.

## Competing Interests

 The authors have no relevant financial or non-financial interests to disclose.

## Ethical Approval

 All assessments were conducted in accordance with ethical principles and under the supervision of the University’s Ethics Committee (Ethic NO. IR.BMSU.BLC.1402.009).
